# Supplementary material for: Plastid Genome Evolution of Two Colony-Forming Benthic Ochrosphaera neapolitana Strains (Coccolithales, Haptophyta)
Source: Int J Mol Sci. 2023 Jun 22;24(13):10485. doi: 10.3390/ijms241310485 (PMC10341766; doi:10.3390/ijms241310485)
Supplement: Supplementary file 1 [file Supplementary Figures.pdf › Supplementary Figures.pdf]

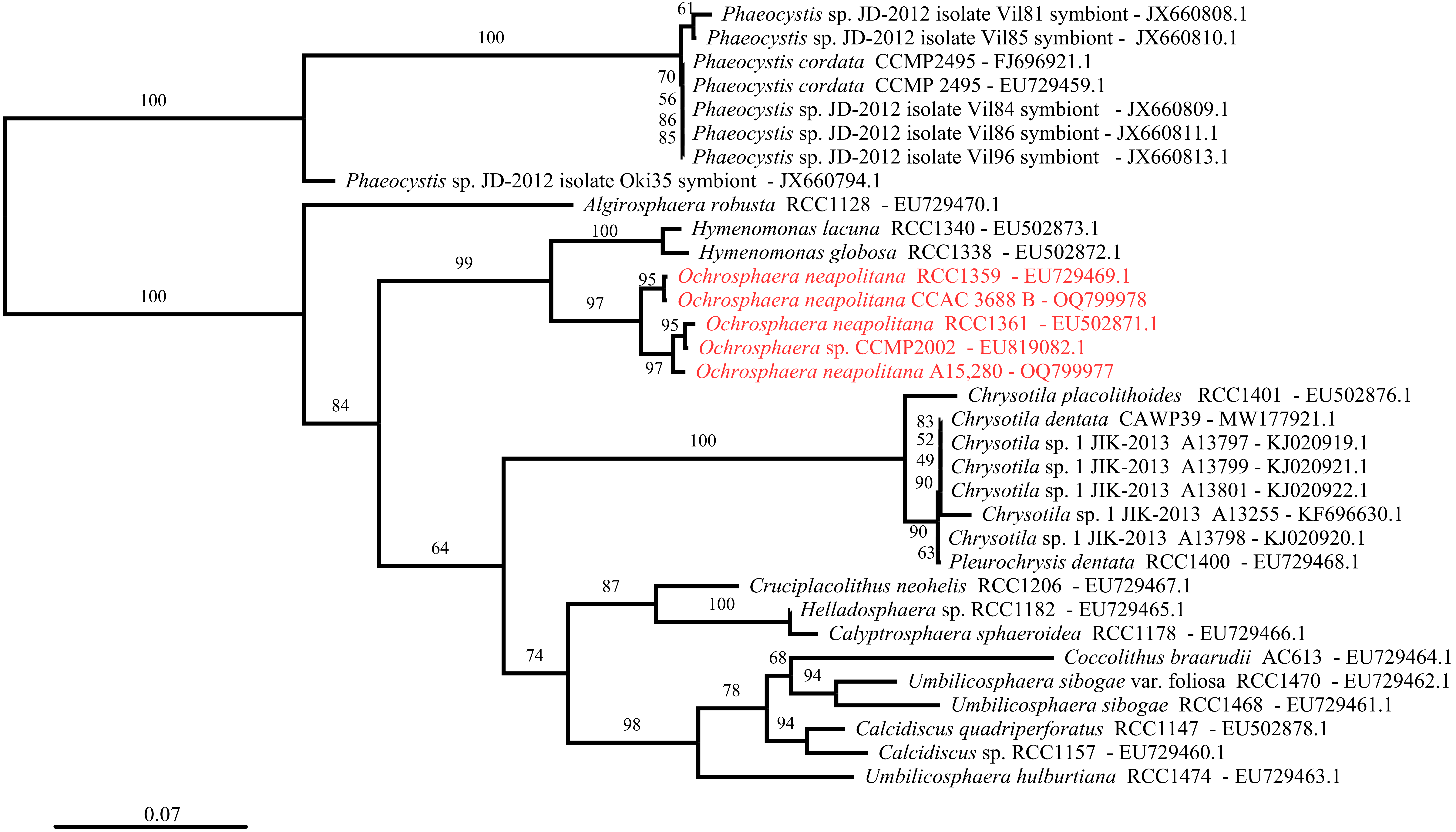


**Supplementary Figure S1.** A haptophyte maximum likelihood phylogenetic tree based on partial 28S rRNA gene sequences.

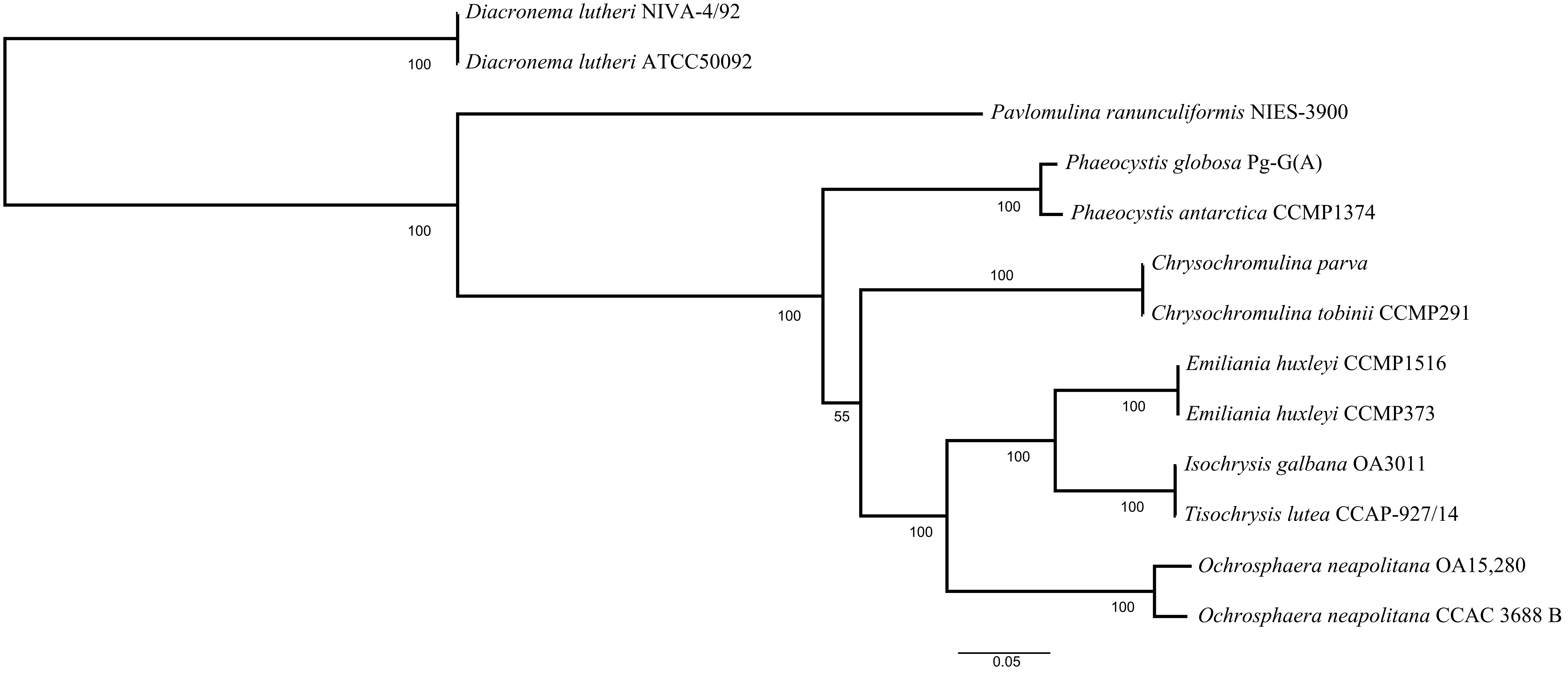


**Supplementary Figure S2.** A ML tree constructed with 88 concatenated single-copy genes from haptophyte plastid genomes.

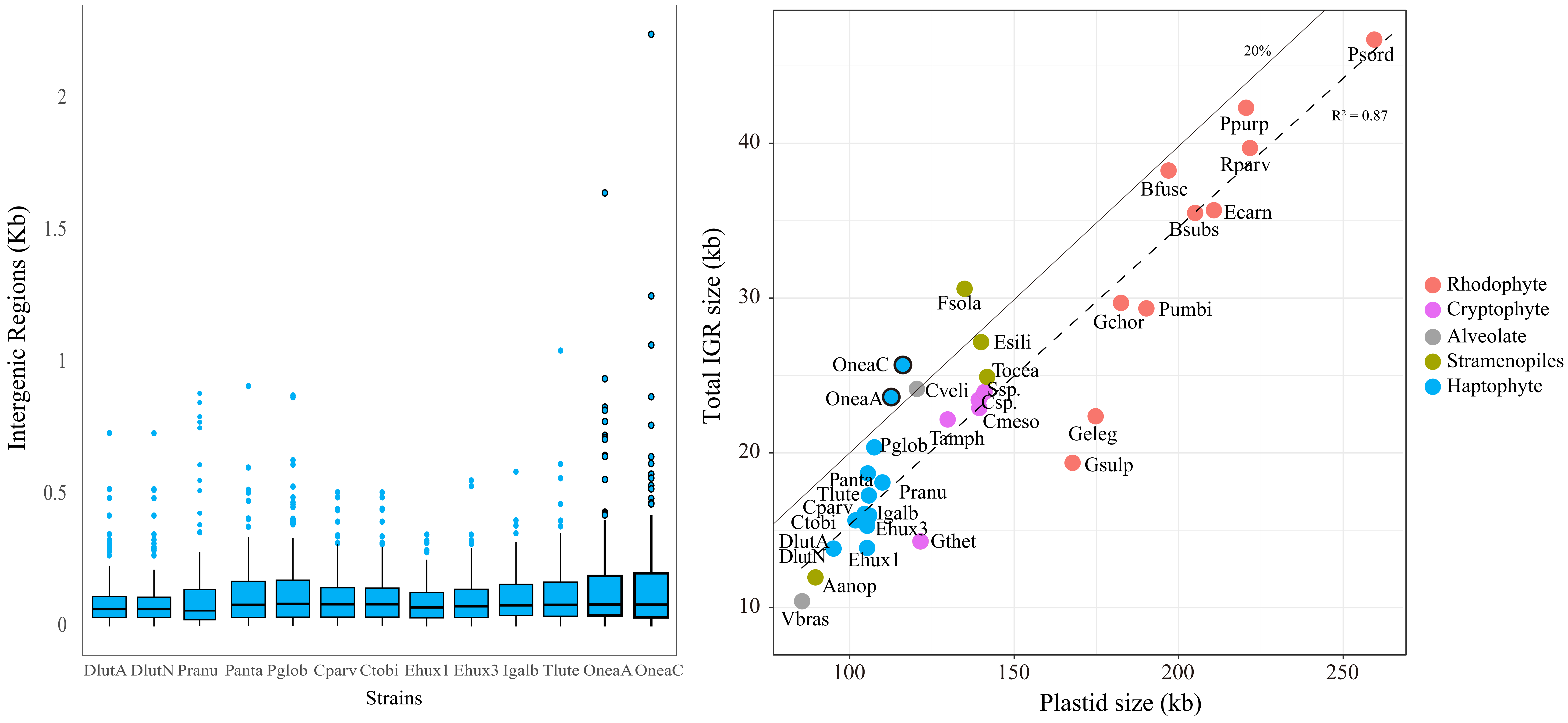


**Supplementary Figure S3.** Average intergenic region size for haptophytes (left panel) and the relationship between total intergenic region size and plastid genome size (right panel) for rhodophytes and CASH lineage organisms. Abbreviations for the strains can be found in Supplementary Table 2.

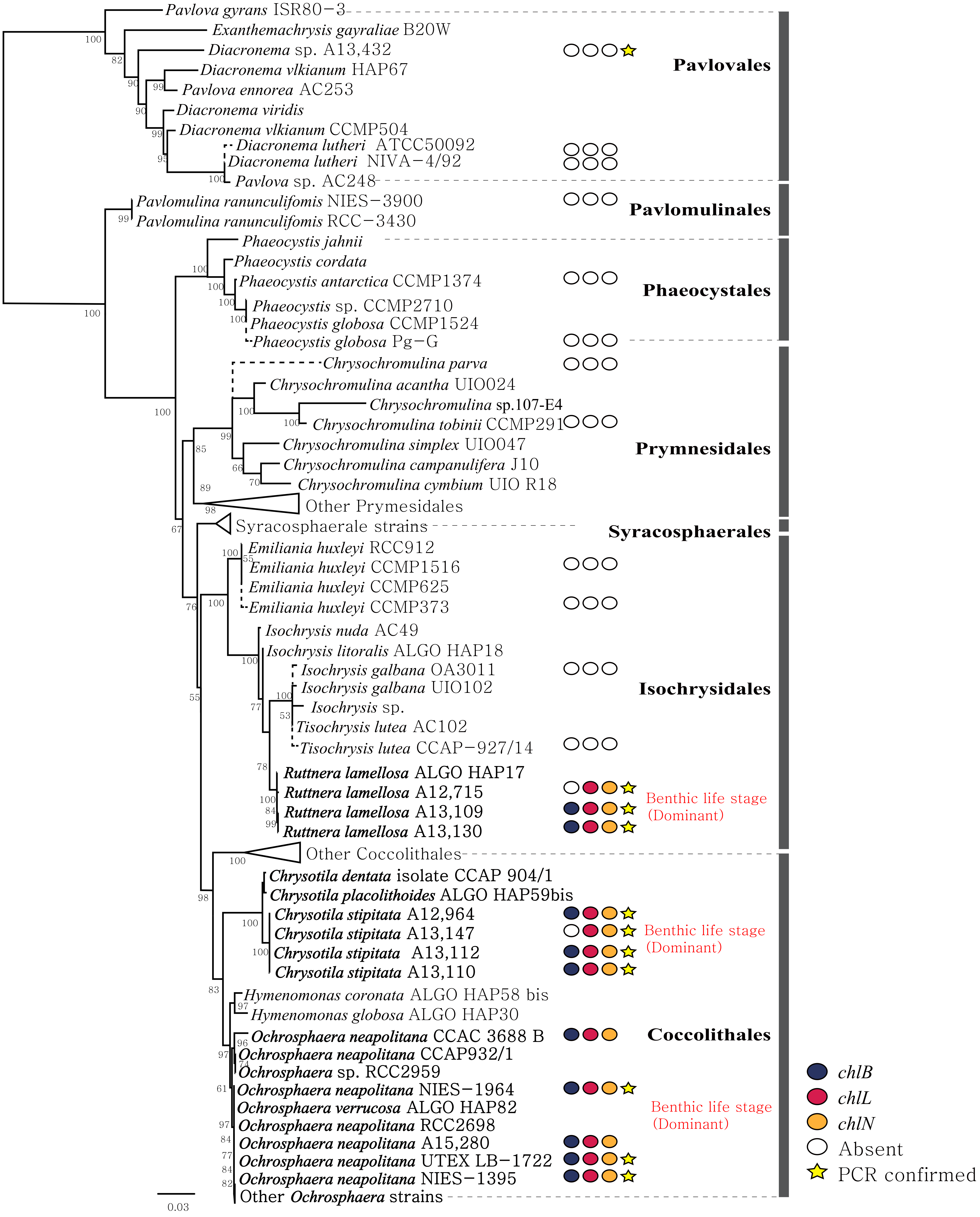


**Supplementary Figure S4.** Distribution of LIPOR genes (colored circles) among haptophytes mapped onto an 18S rRNA phylogeny. In cases where plastid information is accessible but 18S rRNA sequence information is unavailable, an arbitrary dotted line has been used within the phylogenetic tree for graphical representation. Asterisks designate strains where the genes were manually identified using PCR, whereas the taxa in bold were known as having a dominant benthic stage (see Supplementary Table 3).

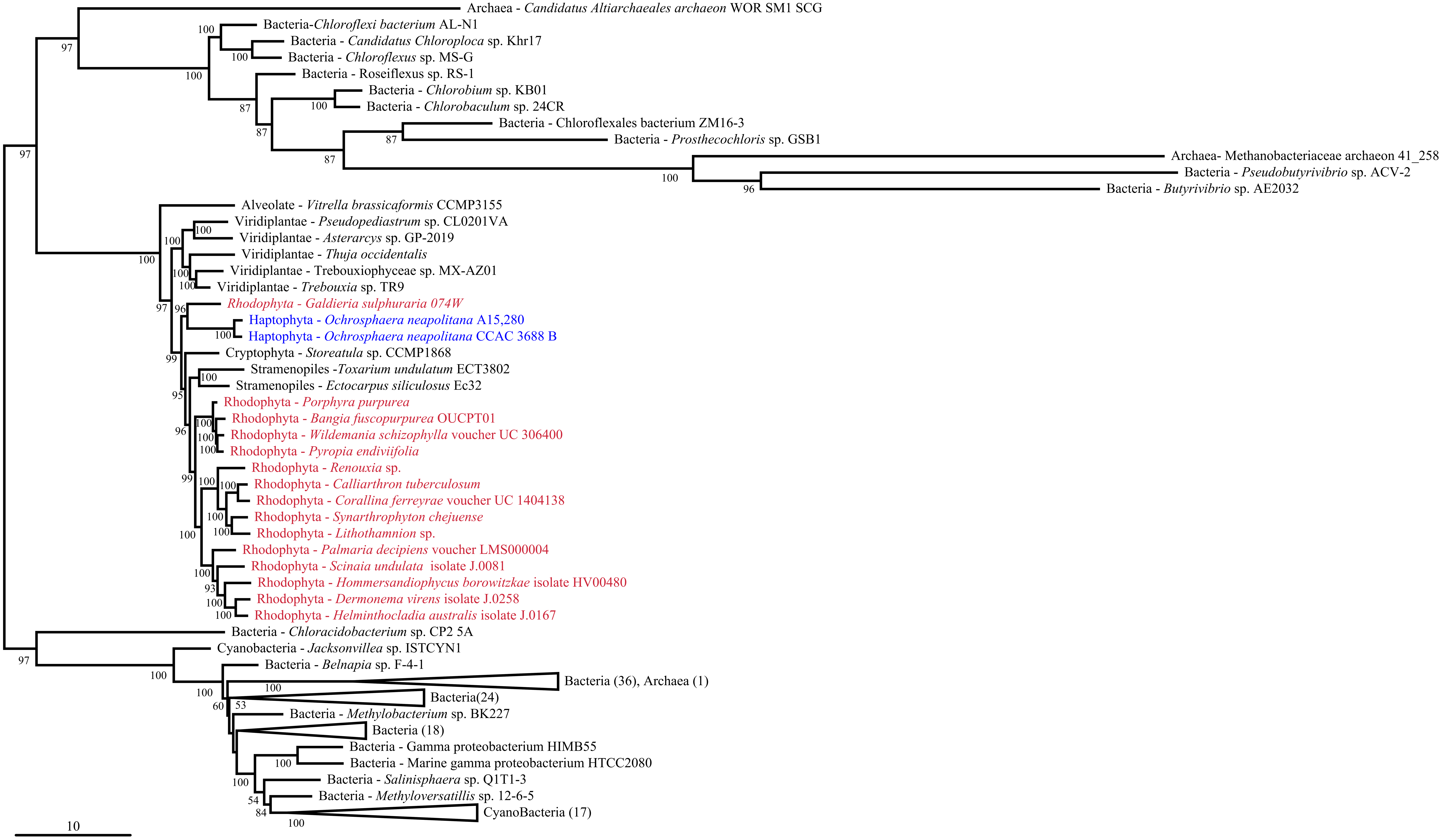


**Supplementary Figure S5.** ML tree based on three concatenated LIPOR genes.

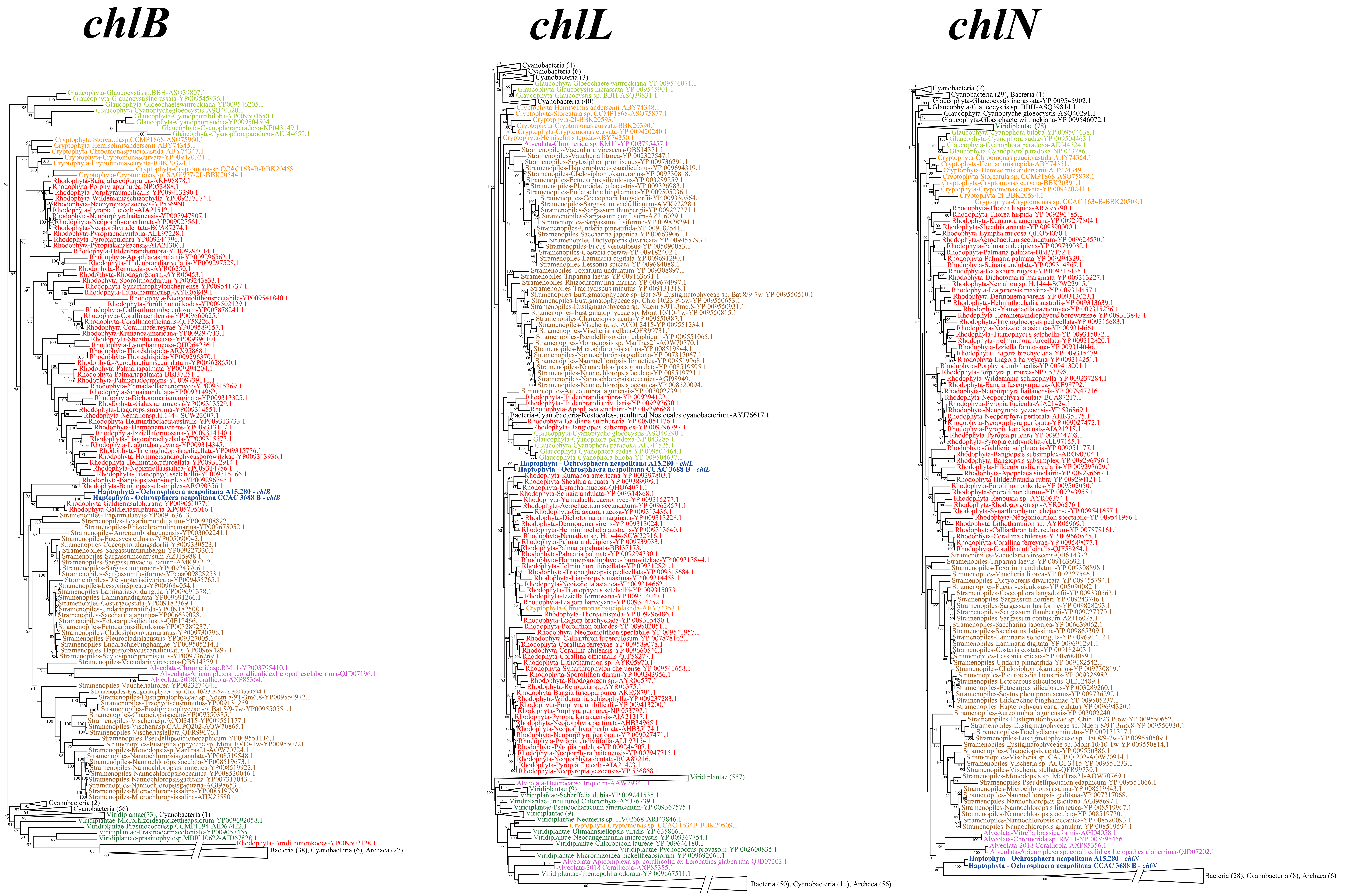


**Supplementary Figure S6.** Individual gene trees for the LIPOR genes (chlB, chlL and chlN). Each color represents a phylum (Blue: Haptophyta, Orange: Cryptophyta, Red: Rhodophyta: Purple: Alveolata, Brown: Stramenopiles, Light green: Glaucophyta, and Green: Viridiplantae).

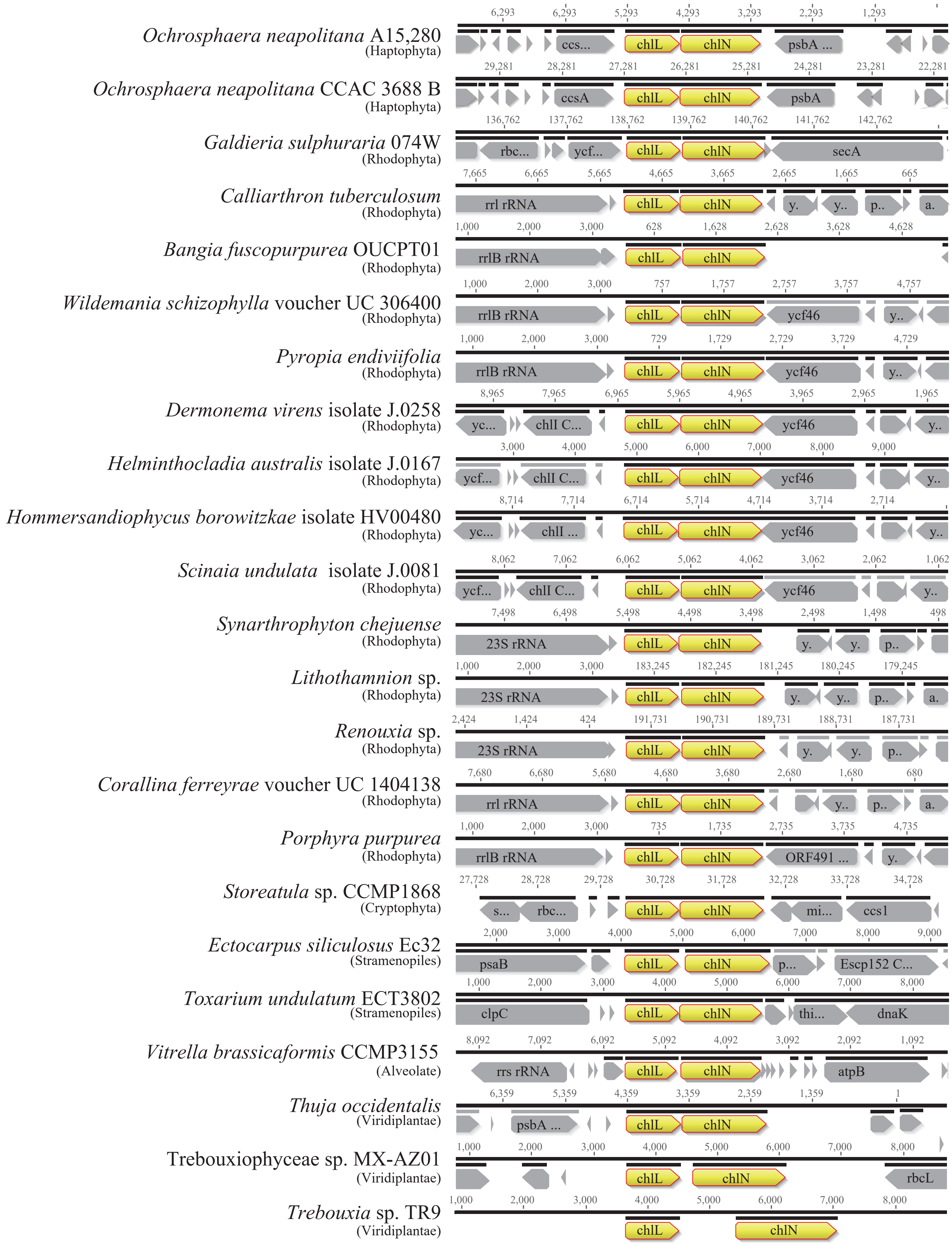


**Supplementary Figure S7.** Synteny block comparison of the chlL-chlN genes from red algae, CASH secondary-plastid groups (cryptophytes, stramenopiles, alveolates, and haptophytes),  green algae and plants
